# Emergent Constraints on Climate-Carbon Cycle Feedbacks

**DOI:** 10.1007/s40641-019-00141-y

**Published:** 2019-09-10

**Authors:** Peter M. Cox

**Affiliations:** College of Engineering, Mathematics and Physical Science, University of Exeter, Exeter, UK

**Keywords:** Carbon cycle, Climate change, Emergent constraints

## Abstract

**Purpose of Review:**

Feedbacks between CO_2_-induced climate change and the carbon cycle are now routinely represented in the Earth System Models (ESMs) that are used to make projections of future climate change. The inconclusion of climate-carbon cycle feedbacks in climate projections is an important advance, but has added a significant new source of uncertainty. This review assesses the potential for emergent constraints to reduce the uncertainties associated with climate-carbon cycle feedbacks.

**Recent Findings:**

The emergent constraint technique involves using the full ensemble of models to find an across-ensemble relationship between an observable feature of the Earth System (such as a trend, interannual variation or change in seasonality) and an uncertain aspect of the future. Examples focussing on reducing uncertainties in future atmospheric CO_2_ concentration, carbon loss from tropical land under warming and CO_2_ fertilization of mid- and high-latitude photosynthesis are exemplars of these different types of emergent constraints.

**Summary:**

The power of emergent constraints is that they use the enduring range in model projections to reduce uncertainty in the future of the real Earth System, but there are also risks that indiscriminate data-mining, and systematic model errors could yield misleading constraints. A hypothesis-driven theory-led approach can overcome these risks and also reveal the true promise of emergent constraints—not just as ways to reduce uncertainty in future climate change but also to catalyse advances in our understanding of the Earth System.

## Introduction

The General Circulation Models (GCMs) used to make projections of future climate change are vitally important to inform climate mitigation and adaptation strategies, but they are also invaluable tools for testing hypotheses about the functioning of the Earth System. GCM projections appear prominently within each of the assessment reports of the Intergovernmental Panel on Climate Change (IPCC). Climate modelling centres around the world have devoted increasing effort to improving GCMs over the quarter of a century since the first IPCC report in 1990. This has led to increases in spatial resolution, improved process representation and the inclusion of new feedbacks. The latter includes the inclusion of carbon cycle feedbacks in GCMs—which ultimately resulted in the evolution of climate models into Earth System Models (ESMs).

As a result of these many ongoing model improvements, ESMs now provide a more complete representation of the myriad of interactions and feedbacks that determine how the climate will change in response to human and natural forcing factors. Unfortunately though, the range of model projections has not significantly reduced despite these improvements. To give a simple example, the projected range of global warming by 2100 still varies by a factor of more than two across the model range, even under a common emission scenario [1]. This uncertainty in climate projections is in large part due to continuing uncertainty in physical climate feedbacks (e.g. feedbacks from clouds, water vapour and ice-albedo) which leads to a large range in the sensitivity of climate to changes in carbon dioxide, other greenhouse gases and aerosols. However, in emission-driven ESM runs, there is also a significant additional uncertainty due to different representations of climate-carbon cycle feedbacks, which lead to differing projections of future carbon dioxide concentrations in the atmosphere. For example, under the RCP8.5 emissions, the projected carbon dioxide concentration by 2100 varies by more than 300 ppmv [2].

ESM development has traditionally been a rather reductionist bottom-up process, with each component of an ESM (e.g. convection, land surface, clouds) being worked upon by a subset of the scientists in a climate modelling centre, before the improved sub-components are coupled together to form a new ESM. The historical simulations of the resulting ESM are then evaluated against observations of the real climate system, to assess the realism of the model. The difficulty with this approach to model evaluation is that it does not focus specifically on the variables in the contemporary observations which are most relevant to the future climate. ESMs are designed to make reliable projections of the future, so it would be preferable to judge the reliability of a given model in terms of the aspects of the contemporary climate that are most relevant to those projections. The concept of emergent constraints is a very promising way to identify the most relevant aspects of climate for future projection [3] and also to derive constrained estimates of key feedbacks in the Earth System [4]. This review focusses specifically on possible emergent constraints on climate-carbon cycle feedbacks.

### Climate-Carbon Cycle Feedbacks

The ocean and land carbon cycles are currently performing an important role for humanity, as they are collectively absorbing about half of global CO_2_ emissions from human activities—the so-called airborne fraction (AF). This means that atmospheric CO_2_, and therefore global temperature, is increasing about half as fast as it would be in the absence of these land and ocean carbon sinks. There are a number of well-known mechanisms that lead to net carbon uptake by land and ocean as the atmospheric CO_2_ concentration increases. Carbon dioxide dissolves in seawater, so more atmospheric CO_2_ ultimately leads to more carbon storage in the ocean. A carbon sink in vegetation and soils on the land can arise from increased plant growth that could be due to enhanced photosynthesis under elevated CO_2_, nitrogen deposition, or high-latitude warming; or from forest regrowth on formerly deforested areas.

The processes underlying these land and ocean carbon sinks are however also known to be dependent on climate. In the oceans less CO_2_ dissolves in warmer seawater. Warming of the ocean surface also suppresses vertical mixing, which hinders the transfer of anthropogenic carbon to depth and may deny nutrients to the phytoplankton that drive the biological carbon pump. On the land respiration fluxes from vegetation and soil, which return CO_2_ to the atmosphere, increase with warming. The distribution and functioning of vegetation also depend strongly on patterns of temperature and rainfall.

As a result of these climate-dependent processes land and ocean carbon sinks, and therefore the airborne fraction of anthropogenic emissions, are sensitive to climate. This is especially evident for the land which switches to become a carbon source during strong El Niño events as a consequence of warming and drying in the tropics. An increase in atmospheric CO_2_, which results in a climate change via the greenhouse effect, may therefore change the carbon storage in the ocean and on land and therefore modify the CO_2_ concentration of the atmosphere producing a further change in climate. We describe this as a climate-carbon cycle feedback.

### Uncertainties in Climate-Carbon Cycle Projections

The extent to which the natural carbon cycle lessens the climate impact of our CO_2_ emissions can be thought of in terms of the airborne fraction (AF), which is the rate of increase of atmospheric CO_2_ concentration divided by the rate of release of CO_2_ by anthropogenic emissions. The combined effect of land and ocean carbon sinks has kept the mean AF at around 0.5 since the mid-nineteenth century. A similarly near-constant AF was assumed into the future for climate projections made prior to the development of ESMs in the late 1990s. As a result, climate model projections up to that time did not account for climate-carbon cycle feedbacks. The first published projection which included the carbon cycle as an interactive element within GCM climate models showed an alarming tendency for the AF to increase through time leading to an acceleration of global warming, mainly due to a widespread warming-induced release of soil carbon and a drying-induced dieback of the Amazonian rainforest [5, 6].

At around this time, a team at IPSL in France was also carrying out their first climate-carbon cycle projections [7]. They also found a positive (i.e. amplifying) climate-carbon cycle feedback, but of a smaller magnitude. Collaboration and comparison of the two model projections revealed that the primary differences were in the response of the land biosphere to climate change. The Cox et al. [5] model projected a more negative impact of climate change on land-carbon storage, most likely due to greater drying in Amazonia, the inclusion of vegetation dynamics and the use of a single soil carbon pool [8].

These differing results motivated an international group to set up the Coupled Climate-Carbon Cycle Model Intercomparison Project (C^4^MIP). The purpose of C^4^MIP was to encourage other climate modelling centres to include an interactive carbon cycle and to coordinate the comparison of climate-carbon cycle projections. The first-generation C^4^MIP models agreed that climate effects on the carbon cycle would accelerate the increase in atmospheric CO_2_. However, the size of this effect varied by an order of magnitude across the models, from 30 to 300 ppmv of extra CO_2_ by 2100 under a common scenario [9].

Largely as a consequence of the C^4^MIP project, many of the climate projections reported in the Intergovernmental Panel on Climate Change (IPCC) 5th Assessment Report (AR5) include climate-carbon cycle feedbacks [10]. While the evolution of the global ocean carbon sink and its large-scale spatial pattern was found to be similar amongst the models, the future land carbon sink had a huge range. For example, even under an idealized scenario of a 1% increase in CO_2_ per year, the projected impact on global land carbon storage varied by more than 500 PgC by 2100 [10] (where 1 PgC *=* 1 billion tonnes of carbon). The divergence amongst model projections was even more significant when feasible changes in land use were included [11].

**Fig. 1:**
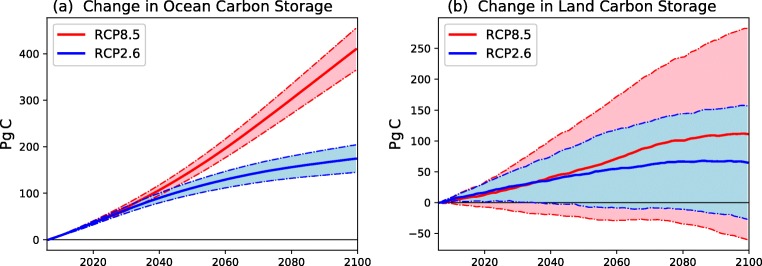
Projected change in **a** ocean carbon storage and **b** land carbon storage for 2005 to 2100 in concentration-driven RCP2.6 (blue) and RCP8.5 (red) scenarios. Thick lines represent the ensemble mean of the available ESM runs, and the shaded area represents ± one standard deviation about that mean

Early results suggest that a similarly large range is to be expected from the CMIP6 projections to be included in the IPCC 6th Assessment Report (AR6). Figure 1 shows the projected range of changes in ocean and land carbon storage in concentration-driven RCP2.6 and RCP8.5 scenarios. The colour wedges represent the ensemble mean plus and minus one standard deviation across the ensemble. For each scenario, the model runs prescribe the same changes in atmospheric greenhouse gas concentrations and land-use change. As for the AR5, the range is very much larger for land than for ocean.

All ESMs predict a declining global ocean carbon sink once the CO_2_ concentration starts to decline from the mid-2020s onwards under RCP2.6, such that the projected ocean sink is around 0.8 ± 0.35 PgC year^−1^ by 2100 (Fig. 1a). Despite increasing CO_2_ under RCP8.5 the ocean carbon sink saturates by 2100 at 5.5 ± 0.7 PgC year^−1^. The change in ocean carbon storage from the present day until 2100 is very different between RCP8.5 (410 ± 45 PgC) and RCP2.6 (175 ± 30 PgC). Once again, we see a much larger range in future projections of the land carbon sink (Fig. 1b). The model ensemble projects a change in land carbon storage from 2005 to 2100 of 65 ± 90 PgC under RCP2.6 and 110 ± 170 PgC under RCP8.5. Even the sign of the change in land carbon storage, which depends on the difference between the net carbon uptake by existing vegetation and net carbon release from land-use change, is therefore disputed.

Coupled climate-carbon cycle models enable contemporary policy-relevant questions to be more directly addressed, such as ‘how much CO_2_ can we emit and still avoid 1.5K of global warming?’*.* Such large uncertainties therefore undermine the value of these projections to inform climate policy. One way to alleviate this problem is by using emergent constraints to reduce key uncertainties in the myriad of interactions between the carbon cycle and climate change.

### The Emergent Constraint Concept

The usual way for the climate modelling community to deal with a spread amongst projections is to look at the performance of each model compared with contemporary observations and then assume the most realistic models produce the most believable projections. This approach has been rather unkindly compared to a beauty contest, as the metrics of model quality are not necessarily relevant to the value of a model for making predictions. Partly as a result of this, it has been difficult to get agreement amongst modelling groups on the choice of model evaluation metrics.

Fortunately, there is another way. Complex Earth System Models (ESMs) simulate variations on timescales from hours to centuries—so in principle, ESMs tell us how aspects of the current observable Earth System relate to its sensitivity to anthropogenic forcing. In the context of this review, variations include historical trends, interannual variability, seasonal cycles or trends in seasonal cycles. Sensitivity relates to a predicted change in the future in response to some given change in forcing (e.g. a change in future atmospheric CO_2_ in response to a given scenario of CO_2_ emissions, or a change in tropical land carbon storage in response to a change in global mean temperature).

We expect different ESMs to often agree on the relationship between variability and sensitivity for two main reasons: (a) short-term and long-term changes are often linked by conservation laws (e.g. relationships between changes in carbon fluxes and changes in carbon stores) and (b) there are theoretical reasons (e.g. the fluctuation-dissipation theorem) to expect variability and sensitivity to be linked in a large class of systems. The fluctuation-dissipation theorem (FDT) links the sensitivity of a linear system to external forcing, to the internal fluctuations of that system. Broadly speaking, more sensitive systems also have fluctuations that are less effectively damped, such that fluctuations are larger and longer-lived. FDT-type ideas were used in statistical thermodynamics even before the FDT was formally defined or proven, most notably by Einstein in his work relating Brownian motion to the diffusivity of a gas. Although the FDT only applies approximately to the climate system [12], it provides the theoretical motivation for a number of emergent constraint studies [13, 14]. In essence, these emergent constraints hypothesise relationships between sensitivity and variability across a model ensemble. Similar ideas are also applied to explain tipping point precursors—trends in system variability as an abrupt transition are approached. In that case, a temporal change in system variability (such as ‘critical slowing down’) reveals a reducing resilience of the system to perturbations that can be extrapolated in time to estimate when a tipping point will occur [15]. The link between tipping point precursors, emergent constraints and the FDT is a promising avenue for future research.

Where an ensemble of different ESMs do agree on a relationship between a short-term observable variation and a longer-term sensitivity, an observation of the short-term variation in the real world can be converted, via the model-based relationship, into a constraint on the sensitivity—called an emergent constraint. An emergent constraint is a relationship between a predicted aspect of the future Earth System and an observable feature of the Earth System, evident across an ensemble of models. Whereas the spread amongst ESM projections is normally considered to increase uncertainty about the future, emergent constraints make use of this spread to make inferences about the sensitivity of the real Earth System. Emergent constraints therefore also offer a means to assess models on the basis of the metrics that are most relevant for the fidelity of future projections.

The term ‘emergent constraint’ was first coined in the context of climate projections by Allen and Ingram [16]. However, the archetypal emergent constraint was demonstrated by Hall and Qu [17], who found a linear relationship between the size of the snow-albedo feedback in climate models and the sensitivity of the seasonal snow cover to seasonal temperature variations in the same models. This relationship was first shown for the generation of models used in the IPCC 4th Assessment (CMIP3) and has since been shown to also hold for the more recent (CMIP5) models. The observational constraint on the seasonal snow cover sensitivity comes from satellite observations of snow cover and observed near-surface temperatures. Broadly speaking, it is possible to convert the range of possible values for this short-term sensitivity into a range of possible values for the snow-albedo feedback, using the across-model relationship between these two variables. This implies an emergent constraint on the snow-albedo feedback in the real world.

The Hall and Qu [17] example has motivated many others to search for emergent constraints on other uncertain aspects of future climate change, including sea-ice loss [18], tropical precipitation extremes [19], cloud feedbacks [20] and many attempts to constrain equilibrium climate sensitivity [21]. It has also encouraged a number of recent studies aiming at finding emergent constraints on the carbon cycle components of the Earth System.

### Examples of Emergent Constraints on Carbon Cycle Feedbacks

**Fig. 2:**
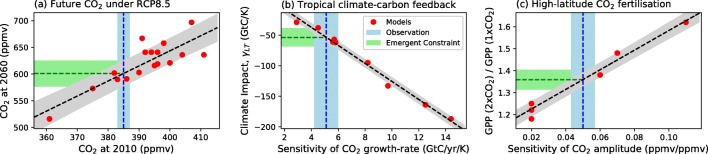
Examples of emergent constraints on the carbon cycle in ESMs. **a** Projected global mean atmospheric CO_2_ concentration by 2060 under the RCP8.5 emission scenario against the simulated CO_2_ concentration in 2010 [2, 22]. **b** Sensitivity of tropical land carbon to temperature increase against the sensitivity of the atmospheric CO_2_ growth rate to tropical temperature variability [23, 24]. **c** Sensitivity of gross primary production (GPP) to a doubling of atmospheric CO_2_ against the sensitivity of the amplitude of the CO_2_ seasonal cycle at Point Barrow, Alaska, to a change in global mean atmospheric CO_2_ concentration [25]

The simplest type of emergent constraint is a relationship between a past-to-present change and a present-to-future change. For example, a relationship was noted in ESM runs between the simulated CO_2_ concentration by 2010 and the projected CO_2_ concentration in the future under the RCP8.5 scenario [2]. As the CO_2_ concentration in 2010 is known from observations, there is a potential emergent constraint on the future CO_2_ concentration under this common scenario [22], as shown in Fig. 2a. Such trend-on-trend relationships depend on models being similarly forced (in this case by CO_2_ emissions consistent with RCP8.5).

Other emergent constraints assume relationships between short-term variability and long-term sensitivity to forcing. The first published emergent constraint on the carbon cycle was of this type, relating to the long-standing problem of the sensitivity of tropical forests to climate change [23]. The amount of carbon released per degree of warming in the tropics spanned a factor of more than four in the C^4^MIP projections (from 29 to 133 GtC/K), ranging from a relatively small effect which was more than counteracted by CO_2_ fertilization to projections involving catastrophic dieback of the Amazon rainforest [5, 6].

There are no direct measurements of changes in tropical land carbon storage to provide the observational constraint, but the year-to-year variation in atmospheric CO_2_ provides a valuable proxy. The annual growth rate of atmospheric CO_2_ is known to be strongly correlated with the El Niño Southern Oscillation (ENSO) predominantly through its climatic impact on the tropical land carbon cycle. By combining observational records for the annual CO_2_ concentration and the annual mean temperature in the tropics, it is possible to calculate the sensitivity of the annual CO_2_ growth rate to interannual (IAV) temperature variability in the tropics. Identical calculations can be undertaken to derive the IAV sensitivities of the CO_2_ growth rate for each of the C^4^MIP models.

A strong linear emergent relationship was found between the IAV sensitivity of the CO_2_ growth rate and the key unknown—the century-timescale sensitivity of tropical land carbon to warming in the tropics (Fig. 2b). By combining this emergent relationship with the estimated IAV sensitivity of the CO_2_ growth rate, it was possible to obtain an emergent constraint on the sensitivity of tropical land carbon to warming of 53 ± 17 GtC/K, compared with the unconstrained C^4^MIP model range of 29–133 GtC/K [23]. A broadly consistent emergent constraint on tropical carbon loss due to climate change has also been derived from the more recent CMIP5 models [24]. In a similar way, satellite estimates of the interannual variability in ocean productivity have been used to constrain projected changes in tropical marine productivity under long-term warming [26].

Other constraints relate the seasonal cycle [17], or changes in the seasonal cycle [25], to the strength of particular feedbacks. A recent carbon cycle example uses the well-documented increase in the amplitude of the seasonal cycle of atmospheric CO_2_ as measured at Mauna Loa and Point Barrow [27] to constrain CO_2_ fertilization of photosynthesis on mid- and high latitude [25], as shown in Fig. 2c. A more complete list of proposed emergent constraints, including on climate-carbon cycle feedbacks, is given in Table 1 of Hall et al. [3].

### Promise, Dangers and the Need to Be Theory-Led

There are many attractive features of emergent constraints, but perhaps the most beguiling is that they offer the possibility of using the continuing range in model projections to reduce uncertainty in the future of the real climate system. This is in stark contrast to the usual interpretation of the projection range, which is as a measure of our uncertainty about the future.

Emergent constraints require both internal consistency of models, so that the observable and future changes are related across the ensemble, and also a significant range of model projections, so that this emergent relationship is well-defined. Emergent constraints therefore make best use of the current situation in climate modelling, where we have an increasing number of complex internally consistent ESMs, but an enduring range of climate projections. It is however rather ironic that the emergent constraint approach, which is designed to reduce model spread, needs model spread in order to be effective. Hall et al. [3] relate this to a Douglas Adams-like ‘emergent constraint paradox’—emergent constraints will cease to exist as soon as model developers start to take them seriously (such that they begin to tune their models to satisfy emergent constraints).

There are dangers associated with the emergent constraint approach as most effectively pointed out by Caldwell et al. [28]. Modern ESMs predict an increasing number of variables on increasingly high-resolution grids. This implies huge numbers of potential output variables, and a risk that indiscriminate data-mining of the multidimensional outputs from ESMs could lead to spurious correlations [28] and proposed constraints on future changes that are not robust [29]. Where the models share similar systematic biases, or neglect similar processes, even statistically significant emergent relationships may mislead about the likely changes in the real system.

Hall et al. [3] suggest an emergent constraint ranking system to assess the robustness of emergent constraints, ranging from ‘proposed’ to ‘confirmed’—once an emergent constraint has both a plausible physical mechanism and has been validated ‘out of sample’ on a model ensemble other than the one it was developed for (e.g. shown to exist for both CMIP5 and CMIP6). To guard further against spurious constraints, Hall et al. [3] also propose a hypothesis-driven approach to testing for emergent constraints. This involves hypothesising a relationship between an observable trend or variation of the system and a projected future change and then checking that relationship across the model ensemble. This theory-led approach would both guard against the risks of indiscriminate data mining and also encourage efforts to understand the Earth System in terms of simpler theoretical models.

In the case of changes in relatively fast variables (such as marine phytoplankton concentration), there may be a fairly straightforward near one-to-one relationship between the short-term variability and the longer-term sensitivity, because the fast variable will be in a quasi-equilibrium state even with short-term climate variations [26]. For slower variables (such as the forest carbon storage), short-term variations are more likely to measure fluxes (or equivalently the rate of change of the store). In this case, finding a constraint on future changes in the store requires multiplying the flux sensitivity to short-term variations by a characteristic timescale for each model. In some cases, the characteristic timescale may be similar across the model spectrum, leading to a simple emergent relationship between the short-term flux sensitivity and the long-term sensitivity of the store [23]. In general though, converting a flux sensitivity to a store sensitivity requires an independent estimate of the characteristic timescale of the store [24].

A recent attempt to develop a theory-guided emergent constraint on equilibrium climate sensitivity [13] was based on analytical solutions to the simplest stochastic energy balance model [30]. The *x*-axis observable in that case was a function of both the variance of the temperature and the autocorrelation of the temperature—which is a measure of the characteristic timescale of the system [14, 31]. There seems to be huge promise in further developing the theory of emergent constraints, including making links to ideas such as the fluctuation-dissipation theorem [12] and the theory of tipping point precursors, such as critical slowing down [15].

Despite the drive towards theory-led emergent constraints, the approach is likely to be semi-empirical for the foreseeable future. Emergent constraints will therefore remain conditional on the model ensembles used to define them and will be subject to systematic biases in the model ensemble. Most obviously, if an important process is neglected in all models (e.g. nutrient limitations on CO_2_ fertilization, or the impacts of forest fires on the interannual variability of CO_2_), this has the potential to lead to spurious emergent constraints on the real Earth System. For this reason, it is vital to reassess emergent constraints with each new generation of models, especially where that new generation includes previously neglected processes and feedbacks.

## Conclusions

The challenge of reducing the spread of model projections has vexed climate science for many decades. Despite great progress in climate modelling and in process understanding, key Earth System sensitivities remain poorly constrained. The emergent constraint approach has been developed to address this impasse by applying a more top-down approach to projections from an ensemble of ESMs. Emergent constraints offer a very attractive way to make the growing number of complex Earth System Models (ESMs) into ‘more than the sum of the parts’. This is achieved by using an ensemble of ESMs to relate an observable trend, interannual variation or change in a seasonal cycle, to a future projected change. The emergent constraint approach is being applied to more and more aspects of the physical climate system [3] and has more recently been applied to feedbacks between CO_2_-induced climate change and the carbon cycle. There have been published studies claiming constraints on carbon loss from the tropics under climate change [23, 24], mid-century atmospheric CO_2_ concentration [22], CO_2_-fertilization of land photosynthesis [25, 32], changes in tropical ocean primary production under warming [26] and permafrost melt [33]. There are recognised dangers which counter the attractive features of emergent constraints—indiscriminate data mining or systematic model errors could lead to spurious conclusions about the real system. A move towards theory-led/hypothesis-driven approach has the promise to mitigate against these risks and also encourage the development of simple models to aid understanding of the real Earth System.
